# Large-scale linkage analysis of 1302 affected relative pairs with rheumatoid arthritis

**DOI:** 10.1186/1753-6561-1-s1-s100

**Published:** 2007-12-18

**Authors:** Marian L Hamshere, Ricardo Segurado, Valentina Moskvina, Ivan Nikolov, Beate Glaser, Peter A Holmans

**Affiliations:** 1 Biostatistics and Bioinformatics Unit and Department of Psychological Medicine, Cardiff University, School of Medicine, Heath Park, Cardiff, CF14 4XN, UK

## Abstract

Rheumatoid arthritis is the most common systematic autoimmune disease and its etiology is believed to have both strong genetic and environmental components. We demonstrate the utility of including genetic and clinical phenotypes as covariates within a linkage analysis framework to search for rheumatoid arthritis susceptibility loci. The raw genotypes of 1302 affected relative pairs were combined from four large family-based samples (North American Rheumatoid Arthritis Consortium, United Kingdom, European Consortium on Rheumatoid Arthritis Families, and Canada). The familiality of the clinical phenotypes was assessed. The affected relative pairs were subjected to autosomal multipoint affected relative-pair linkage analysis. Covariates were included in the linkage analysis to take account of heterogeneity within the sample. Evidence of familiality was observed with age at onset (*p *<< 0.001) and rheumatoid factor (RF) IgM (*p *<< 0.001), but not definite erosions (*p *= 0.21). Genome-wide significant evidence for linkage was observed on chromosome 6. Genome-wide suggestive evidence for linkage was observed on chromosomes 13 and 20 when conditioning on age at onset, chromosome 15 conditional on gender, and chromosome 19 conditional on RF IgM after allowing for multiple testing of covariates.

## Background

Rheumatoid arthritis (RA) is the most common systematic autoimmune disease and is believed to have both strong genetic and environmental components in its etiology. Females are at a higher risk than males and their age at presentation shows considerable variability [1]. Here we describe an analysis of a combined sample of raw genotypes provided by Genetic Analysis Workshop 15 (GAW15), comprising four family-based samples known by their collection center as NARAC (North American Rheumatoid Arthritis Consortium), UK (United Kingdom), ECRAF (European Consortium on Rheumatoid Arthritis Families), and Canada. The aim of the analysis was to investigate the familiality of clinical phenotypes and then employ them and genetic phenotypes as covariates in a linkage analysis framework to allow us to investigate models, such as locus heterogeneity, that give rise to different phenotypes within RA.

## Methods

Autosomal microsatellite data for the NARAC, UK, and ECRAF samples were combined with the Illumina single-nucleotide polymorphism (SNP) sample from Canada. The genotypic data were stored in sample-specific files and their marker maps were aligned to improve map correspondence between samples. The Canada and ECRAF loci were placed on the NARAC and UK genetic map using the NCBI physical positions of NARAC, ECRAF, and Canada loci – see Segurado et al. [2] for more detail. Non-Caucasians were removed from the NARAC sample to minimize heterogeneity. No ethnicity information was available for the UK, ECRAF, and Canada samples. The software GRR [3] was used to identify potentially incorrect inheritance structures within pedigrees. These pedigrees were subsequently removed from further analyses. No Mendelian inheritance errors were detected with PedCheck [4].

### Linkage disequilibrium (LD)

We removed evidence of LD to minimize the chance of excessive false positives [5]. Microsatellite markers separated by less than 0.5 cM were identified and those with the lowest single-point information content were removed. The SNP map was thinned to a 0.5 cM grid on the basis of location. Any remaining SNP pairs with an *r*^2 ^> 0.05 and separated by less than 5 cM were thinned further until no LD remained.

### Phenotypes

The genetic and clinical phenotypes analyzed were gender (binary), age at onset (AAO; continuous), definite erosion (binary), and rheumatoid factor (RF) IgM (four levels; treated as continuous). The ECRAF and Canada phenotype information available was limited to gender and RA status. The RA susceptibility locus *HLA-DRB1 *on chromosome 6 was also investigated. We defined a binary measure for *HLA *to represent whether an individual carried a high risk allele, as described in the GAW15 Problem 2 data description [1]. An individual was coded as HLA+ if they carried at least one copy of the five high risk alleles, i.e., DRB1*0401, 0404, 0405, 0408, or 0409. HLA- was defined as no copies of the seven medium increased risk (i.e., DRB1*0101, 0102, 0104, 0105, 1001, 1402, 1406) or the five high risk alleles.

### Statistical analysis

The familiality of the phenotypes AAO, definite erosion, and RF in the individuals affected with RA was assessed in a mixed-effects regression framework by taking the phenotype of interest as the dependent variable, implemented in the software packages MIXOR [6] and MIXREG [7]. Intra-class correlation coefficients (ICCs) were estimated and indicate the proportion of unexplained variance attributable to family membership, i.e., the strength of the familial effect.

Multipoint model-free affected relative-pair (ARP) linkage analysis was performed with the raw phenotypes AAO, definite erosion, RF, HLA, and gender included as covariates (in separate analyses). Sample-specific allele frequencies and pair-wise IBD (identity-by-descent) allele sharing probabilities using information from the full pedigree were estimated by MERLIN [8] at 2 cM intervals. For each chromosome, the IBD estimates from the four samples were combined into a single file. Assuming the maternal and paternal alleles to be inherited independently, the allele sharing probability, *p*_*r*_, can be modelled in a logistic regression framework and can be written as logit(*p*_*r*_) = *O *+ *α *+ *βx*, where *O *is a fixed offset that depends on the relationship between the pair, *α *is a measure of divergence of IBD from the null in the sample as a whole, and *β *incorporates covariate *x *into the model. Because the parameters *p*_*r*_, *O*, and *α *are based on pairs of individuals, so must be the covariate parameter. When considering a continuous measure, covariates were constructed for the mean and difference for each pair. A binary measure (- or +) was resolved into either -/-, -/+, or +/+ pairs of individuals. For further information on including covariates in the model and constraining the parameters, see Hamshere et al. [9]. The IBD estimates and covariate data were then used to estimate the allele sharing probability *p*_*r*_, given particular covariates, and then to obtain ARP linkage statistics. Because *HLA *resides on chromosome 6, no HLA covariate analysis was performed on chromosome 6.

For each chromosome and covariate, two multipoint LOD scores were produced at each 2-cM position: i) the covariate LOD score and ii) a univariate LOD score, in only the ARPs included in the covariate analysis, i.e., excluding those with missing covariate data. An increase in the maximum LOD score over the chromosome (i–ii; ILOD) in excess of 2.0 was taken to indicate a potential covariate effect. Empirical significance levels for each LOD score peak in the observed data were obtained as follows: 10,000 replicates of chromosome 22 were simulated in the absence of linkage, using the same pedigree structures, marker locations, marker allele frequencies and missing genotype patterns as the original data. The average number of peaks per chromosome reaching the required height was calculated from these replicates (note: peaks were defined as local maxima in the LOD score curve separated by at least 30 cM). The number of peaks per genome was approximated by multiplying by 60 (since the length of chromosome 22 is approximately 1/60 of the total length of the autosomes in this sample). This procedure gives similar results to those obtained by simulating replicates of all 22 chromosomes (data not shown), and is considerably easier computationally. Correction for the multiple testing of six non-independent genome scans was applied as follows. First, criteria were chosen for each covariate to give the same significance level (i.e., number of peaks expected by chance per genome scan) as the test peak. Then, for each replicate chromosome, the locations and heights of all the peaks from all six covariates were combined into a single list, and the total number of peaks greater than their corresponding criterion was obtained. The distance criterion of 30 cM for defining separate peaks ensured that peaks from several covariates that are close together (i.e., non-independence) were counted only once. The expected number of peaks per genome was calculated as before. Following Lander and Kruglyak [10], we called peaks in the observed data "genome-wide significant" if the expected number of peaks per genome at least as high as in the simulated data was ≤ 0.05, and "genome-wide suggestive" if this quantity was < 1.0.

## Results

The sample comprised 1302 ARPs informative for linkage from 982 pedigrees, originating from 633 (466) NARAC, 494 (370) UK, 117 (88) ECRAF, and 58 (58) Canada samples of ARPs (pedigrees). The NARAC and UK samples contributed a total of 61 non-sibling ARPs. Estimates of the ICCs indicate the familiality of each phenotype and are presented in Table 1. We observed genome-wide significant evidence for linkage on chromosome 6. Three other chromosomal regions were identified with a univariate LOD score > 1.0 but did not reach the criteria for genome-wide suggestive linkage; these were found on chromosomes 12, 16, and 18. Linkage covariate analyses produced one, two, and one chromosomes with genome-wide suggestive evidence for linkage when gender, AAO, and RF were included in the model, respectively (see Table 2). Neither the HLA nor definite erosion covariates produced increases in maximum LOD score > 2.0.

**Table 1:** Phenotype descriptive statistics, intraclass correlation coefficients (ICCs)

Phenotype^a^	Mean	SD	*N*	ICC	*p*_ICC_^b^	No. ARPs	No. pedigrees
Gender	0.61	0.49	7213	-	-	1302	982
AAO (years)	39.84	13.51	2405	0.38	<<0.001	1030	786
Definite erosion	0.90	0.30	2063	0.08	0.21	789	630
RF	1.99	1.16	2083	0.18	<<0.001	785	608
HLA	0.76	0.42	2430	-	-	870	662

^a^Binary measures: - or male: 0, + or female: 1.

^b^*p*_ICC _is not corrected for multiple testing.

**Table 2:** Summary of LOD scores of interest

Chr	Covariate	Maximum LOD score (cM)	ILOD^b^	Covariate allele sharing information^a^	No. peaks/genome	No. peaks/genome (6 scans)
1	AAO	2.52 (228)	2.12	earlier	1.467	6.60
6	-	20.73 (46)	-	-	0.000	0.00
9	AAO	3.36 (46)	2.80	later & more similar	0.283	1.37
11	Gender	2.38 (70)	2.04	0.61, 0.44, 0.53	1.267	5.78
12	-	1.36 (48)	-	-	1.467	6.60
13	AAO	3.80 (34)	2.74	earlier	0.124	0.64
15	Gender	3.71 (66)	3.34	0.59, 0.41, 0.50	0.059	0.33
16	-	1.29 (44)	-	-	1.791	8.00
18	-	1.38 (80)	-	-	1.361	6.19
19	RF	4.73 (88)	2.36	more similar	0.012	0.07
20	AAO	3.88 (100)	3.28	earlier	0.112	0.59

^a^For continuous covariate measures, the region of the distribution with increased allele sharing are given. For the gender covariate (M, male; F, female), the M/M, M/F and F/F allele sharing probabilities at the maximum LOD score location are presented.

^b^ILOD, increase in maximum LOD score.

## Discussion

The highly significant clustering of AAO and RF within pedigrees replicates the evidence observed in monozygotic twins [11], suggesting they make good candidates for inclusion in the linkage analysis framework. We do not see similar clustering with definite erosions, although because 90% of the individuals have had a definite erosion, there is little power to detect clustering within families or difference in the allele sharing probabilities.

The maximum LOD score of 20.73 at 46 cM on chromosome 6 is by far the most convincing evidence for linkage that we observe. This peak is at the location of the genetic locus *HLA*, which is thought to contribute 30 to 50% of the total genetic component of RA [12,13]. We also observe evidence for loci on chromosomes 12, 16, and 18, the size of their genetic effect is considerably less than that of *HLA*. Evidence for all four loci was also observed by Etzel et al. [14] and in the extended sample of GAW15 [2], using the SNP instead of microsatellite data where available. There was no evidence observed for any locus × locus interaction effects with *HLA *(tested as a binary covariate). This suggests that the regions on chromosomes 12, 16, and 18, if true linkages, may harbor genes that act independently of *HLA*. Our HLA measure excluded individuals who carried medium but no high risk alleles. Two additional HLA measures were created, where the seven medium risk alleles contribute to 1) the HLA+ group and 2) the HLA- group. The linkage results were unchanged. It is possible that we have lost important *HLA *allelic information when creating our binary HLA measure. Also, if *HLA *is not the actual disease gene, but in LD with a disease-causing mutation of relatively large effect, as suggested by the very large LOD on chromosome 6, a covariate analysis with the binary HLA measure may be less powerful than using a covariate based on the IBD information [15], especially if not all individuals have *HLA *genotypes. To investigate this further we performed covariate analyses conditional on the chromosome 6 IBD information (30 to 80 cM), producing two regions with an ILOD > 2.0, both having chromosome-wide significance (see Table 3). We do not replicate the interaction found by John et al. [16] with chromosome 6 (30 to 120 cM) and chromosome 16 observed in the NARAC and UK samples (chromosome-wide *p *= 0.323).

**Table 3:** Summary of chromosome-wide significant identity-by-descent (IBD) allele sharing interaction LOD scores

Run chromosome	Conditional chromosome	Interaction LOD (run cM, conditional cM)	Chromosome-wide significance	Direction of IBD correlation
3	6	3.66 (208, 52)	0.031	+
12	6	3.55 (120, 34)	0.020	+

A benefit of combining the raw genotypes of samples is that the power to detect disease susceptibility loci of small effect is increased. However, it is possible that pooling samples introduces heterogeneity. Incorporating origin of sample as a covariate in the analysis detected evidence of allele sharing heterogeneity only on chromosome 12 (chromosome-wide *p *= 0.04; IBD estimates of NARAC: 0.46, UK: 0.47, ECRAF: 0.61, Canada: 0.58 at 104 cM), suggesting that heterogeneity is minimal. Each of the covariate regions of interest were analyzed in the separate samples, e.g., NARAC only. All regions showed similar effects in the separate samples as in the combined sample. The inclusion of gender, AAO, and RF as covariates produced some potential regions of interest. We observed evidence for an AAO covariate effect on chromosome 1 (expected to occur by chance 1.467 times per genome scan) within 7 cM of an effect observed by analyzing only NARAC and UK individuals with an AAO < 40 years [16]. Our method of incorporating covariates allows all individuals to be included in the analysis and for a direct comparison with the model without the covariate as they are based on the same sample size. We do not replicate the results for AAO, erosion, or HLA in the ECRAF sample [17] (we do not have access to this phenotype data); both [16,17] analyzed peak regions of interest.

The RF data we analyzed were ordinal (four levels). This was the highest common form of the data as the original data were a combination of ordinal (UK) and continuous (NARAC). We subjected chromosome 19 to further scrutiny by analyzing the NARAC sample with RF as continuous data. We did not replicate the results. The distribution of the continuous RF data is very highly positively skewed, with no obvious outliers. Of the total sample, 45% of the continuous RF data was coded as 4 (high positive) in the ordinal RF measure. We transformed the data with log_10_, log_e_, and square root to produce a distribution more similar to the normal distribution – our linkage results were unchanged. This suggests that i) the distribution of the continuous covariate is not important and ii) the chromosome 19 RF result may be an artifact of creating an ordinal measure from continuous data and analyzing it as a continuous measure.

We also considered two family-based methods that use a single covariate defined for each pedigree: a family-wise ARP method in which the covariate (e.g., minimum AAO) is assigned to all ARPs from that family and an ordered subset analysis (OSA) in which pedigrees are ranked on their covariate values and sequentially added to the analysis with FLOSS [18]. We focus on the AAO phenotype because it was the only measure that presented enough variability in the sample to be suitable for OSA. We considered three covariates, the mean, minimum and maximum AAO, all of which were highly correlated (Pearson *r*, *p *<< 0.001). Family-wise ILODs > 2 were observed on chromosomes 13 and 20, with both mean and maximum (the minimum measures gave ILODs > 1). OSAs also identified these two regions; chromosome 13 (maximum: *p *= 0.0096, based on *n *= 80 pedigrees), chromosome 20 (maximum: *p *= 0.0001, *n *= 146, mean: *p *= 0.0002, *n *= 159; minimum: *p *= 0.0128, *n *= 167). The regions identified with family-wise measures lie within 10 cM and with the same direction of effect as those identified to be genome-wide suggestive (after adjusting for six scans) with the pair-wise measure. The similarity of the results from the three covariate methods is likely to reflect minimal heterogeneity of AAO within pedigrees, corresponding to the highly significant ICC (*p *<< 0.001) presented in Table 1.

## Conclusion

We provide evidence to support the familiality of AAO and RF IgM. Incorporating covariates in the linkage analysis allowed us to identify novel regions that may harbor RA susceptibility loci. This information may be useful in conducting future population based case-control analyses, where it will be important to take into account the particular covariate. Replication in independent samples will be important to determine whether our findings are owing to chance alone.

## Competing interests

The author(s) declare that they have no competing interests.
